# Barriers and Challenges Faced by Social Workers Caring for Dementia Patients in Acute Care Settings

**DOI:** 10.1093/geroni/igaa057.251

**Published:** 2020-12-16

**Authors:** Ruth Dunkle, Katherine Cavagnini, Joonyoung Cho, Laura Sutherland, Helen Kales, Cathleen Connell, Amanda Leggett

**Affiliations:** 1 University of Michigan, Ann Arbor, Michigan, United States; 2 Wayne State University, Detroit, Michigan, United States; 3 UC Davis, Sacramento, California, United States; 4 The University of Michigan, Ypsilanti, Michigan, United States

## Abstract

The nature of dementia care provided by social workers across various hospital settings is unexplored. This study utilized the “rigorous and accelerated data reduction” (RADaR) qualitative analysis technique to explore the process of care among social workers for persons with dementia (PWDs) across a Midwestern tertiary care system with two aims: 1) to identify environmental barriers and supports to quality dementia care in two hospital settings (medical and psychiatric emergency departments (ED), and the main inpatient hospital (IP)), and 2) to identify existing strengths and challenges to high quality social work dementia care within these settings. Twelve qualitative interviews were conducted with a purposive, snowball sample of social workers in dementia care in a large, academic health care system in 2016. Results identify environmental barriers in both settings (physical space design, patient-environment interactions, safety, and discharge disposition). Environmental aspects that promote quality care include supportive staff and family in the patient environment in the IP and ED hospital sections while the discharge disposition is more relevant in the IP. While there are some areas of social work involvement (discharge, psycho-social needs, treatment/management issues) that promote quality of care across locales, the pattern of performing roles varied, e.g. there is more focus on discharge planning and less management of competing demands in the IP than in the ED. Also, social workers were more involved in the diagnosis of dementia in the ED than other settings. We offer policy and practice recommendations to improve care for PWDs in academic hospital settings.

